# Primary diffuse large B-cell lymphoma developing within a rectal tubular adenoma with low-grade dysplasia: a case report

**DOI:** 10.1186/1752-1947-8-103

**Published:** 2014-03-24

**Authors:** Francesco Genovese, Gaspare Becchina, Claudia Nagar, Gabriella Ottoveggio, Benedetto Giacalone, Giuseppa Scaglione, Elisa Varriale, Vincenzo Tralongo

**Affiliations:** 1 Department of Diagnostic Laboratory, U.O.C. of Pathological Anatomy, “G.F. Ingrassia” Hospital, ASP Palermo, Italy; 2 Oncology Unit, “Buon Consiglio Fatebenefratelli” Hospital, Naples, Italy

**Keywords:** Adenoma, Diffuse large B-cell lymphoma, Gastrointestinal lymphoma, Polyp

## Abstract

**Introduction:**

Colorectal lymphomas represent only 5% to 10% of gastrointestinal lymphomas, after the stomach and small intestine. Primary lymphoma of the colon and rectum is an unusual observation, constituting only 0.2% to 0.5% of all malignant tumors arising from the colorectal region. Very little is known about the correlation between adenoma and lymphoma in the colorectal tract. We report here a rare case of diffuse large B-cell lymphoma developing within a solitary tubular adenoma with low-grade dysplasia of the rectum.

**Case presentation:**

An 83-year-old Caucasian man was referred to our hospital intermittent anal bleeding and irregular bowel. Colonoscopy revealed a 1cm solitary rectal polyp, which was completely removed by endoscopic resection. Histologic studies revealed low-grade intraepithelial dysplasia; the stroma of adenoma showed focal localization by highly proliferative lymphoid cells. Immunohistochemical analyses demonstrated that lymphoid cells were positive for CD20 and bcl2, whereas they were negative for CD3, CD5, CD10, CD23, CD30, CD138 and cyclin D1. Approximately 90% of the neoplastic cells reacted positively when stained with an antibody to Ki-67. Molecular studies showed the presence of a monoclonal immunoglobulin heavy chain gene rearrangement.

To determine primary or secondary lymphoma localization, Dawson’s criteria were applied to the case. A diagnosis of primary diffuse large B- lymphoma Ann Arbor stage 1A was established. Subsequently, the patient was referred to oncology to establish the stage and to select appropriate treatment.

**Conclusions:**

The case of diffuse large B-cell lymphoma developing within a tubular adenoma, as reported here, is considered a rare event. Little about the prognosis of primary colorectal lymphomas is available and therapeutic treatment protocol is unclear. This case report provides more information on the history and macroscopic appearance of lymphomas presenting in an unusual location. To report additional cases in the future would be helpful in redefining the diagnostic, prognostic and therapeutic approach.

## Introduction

Colorectal lymphomas constitute only 5% to 10% of gastrointestinal (GI) lymphomas, after the stomach and small intestine. Of these large bowel lymphomas, 60% are located in the caecum, 20% in the rectum, and the remainder throughout the colon.

The most common symptoms in more than half of patients are abdominal pain and weight loss or a change in bowel habits. Lower GI bleeding can be found in 13% to 82% of patients. As with GI malignancies, the initial clinical manifestations and the gross appearance of the lesions appear to be related to the site of GI involvement. Thus, obstructing masses or wall thickening are more frequent in small bowel involvement, whereas polyps are predominant in the colon and rectum.

Most colorectal lymphomas are secondary involvement by widespread diseases. Primary lymphoma of the colon and rectum is an unusual observation, constituting only 0.2% to 0.5% of all malignant tumors arising from the colorectal region with caecum, ascending colon and rectum more often affected. On histological examination, almost 90% of the primary GI lymphomas are of B-cell lineage with very few T-cell lymphomas and Hodgkin lymphoma [1-8]. Although there have been reports regarding primary GI lymphomas alone [9-11] or in association with GI malignancies [12-15] very little is known about the correlation between adenoma and lymphoma in the colorectal tract [16,17]. Here we report a rare case of diffuse large B-cell lymphoma (DLBCL) developing within a solitary rectal tubular adenoma with low-grade dysplasia in an 83-year-old man with immunophenotypical and genetic characterization.

## Case presentation

**Figure 1:**
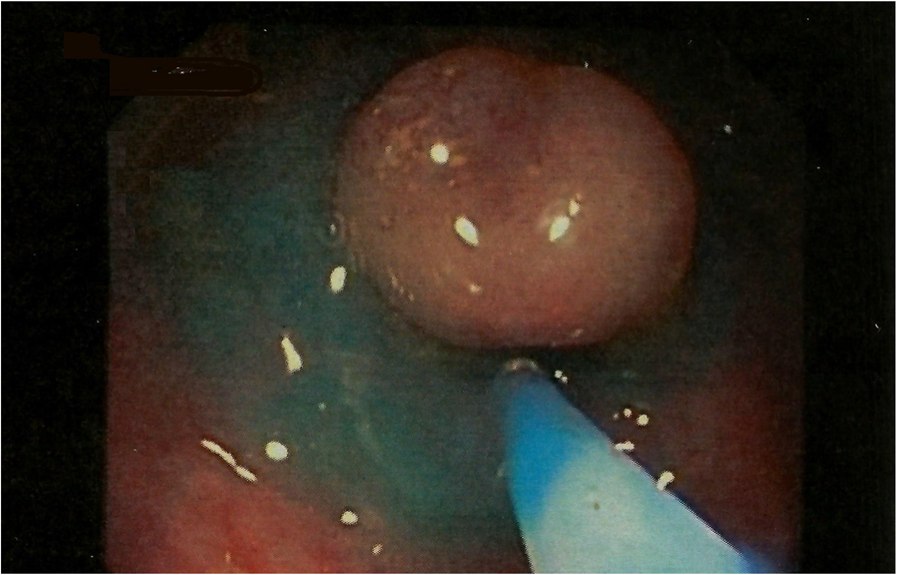
Polyp.

An 83-year-old Caucasian man, in otherwise good health, was referred to our hospital with intermittent anal bleeding and irregular bowel patterns. A colonoscopy revealed a 1cm solitary rectal polyp, which was completely removed by endoscopic resection (Figure 1).

**Figure 2:**
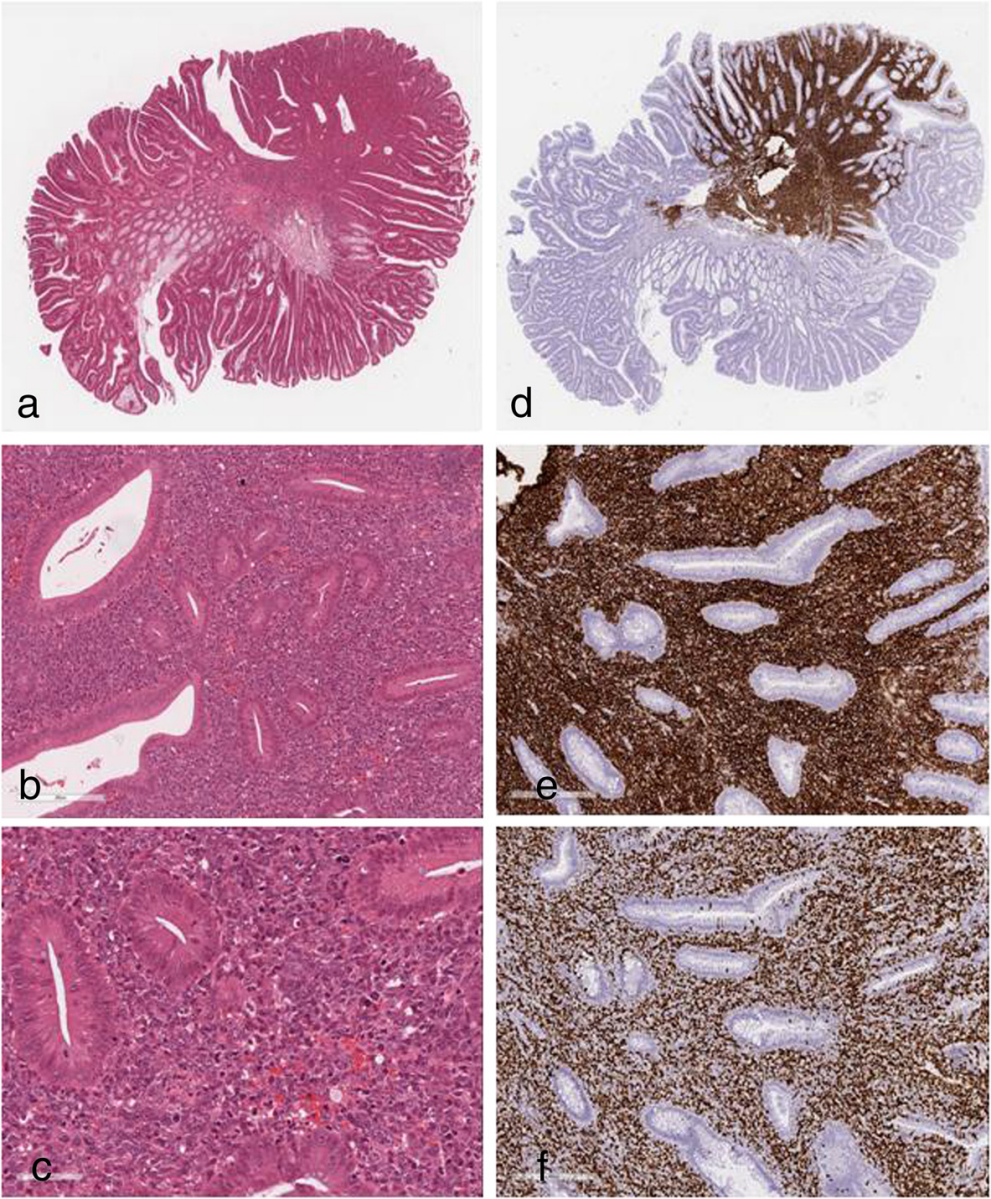
**Histological and immunohistochemical stains. (a)** Polyp histological section, original magnification × 13; **(b)** tubular adenoma with low-grade intraepithelial dysplasia and focal localization in the stroma by highly proliferative large lymphoid cells, hematoxylin and eosin, original magnification × 160; **(c)** and × 400; **(d)** CD20×12; **(e)** and × 100; **(f)** Ki-67×100.

Histological examination showed a tubular adenoma with low-grade intraepithelial dysplasia and focal localization in the stroma by highly proliferative large lymphoid cells (Figure 2a-c). The base of the adenoma was not involved.

Immunohistochemical stains demonstrated that the lymphoid cells expressed CD20 (clone 2B11 + PD7/26, DAKO; Figure 2d and 2e) and B-cell lymphoma 2 (clone 2B11 + PD7/26, DAKO; figure not shown), but not CD3 (clone F7.2.38, DAKO), CD5 (clone 4C7, DAKO), CD10 (clone 56C6, DAKO), CD23 (clone DAK-CD23, DAKO), CD30 (clone Ber-H2, DAKO), CD138 (clone MI15, DAKO) and cyclin D1 (clone EP12, DAKO). Approximately 90% of the neoplastic cells reacted positively when stained with an antibody to Ki-67 (clone MIB1, DAKO; Figure 2f).

**Figure 3:**
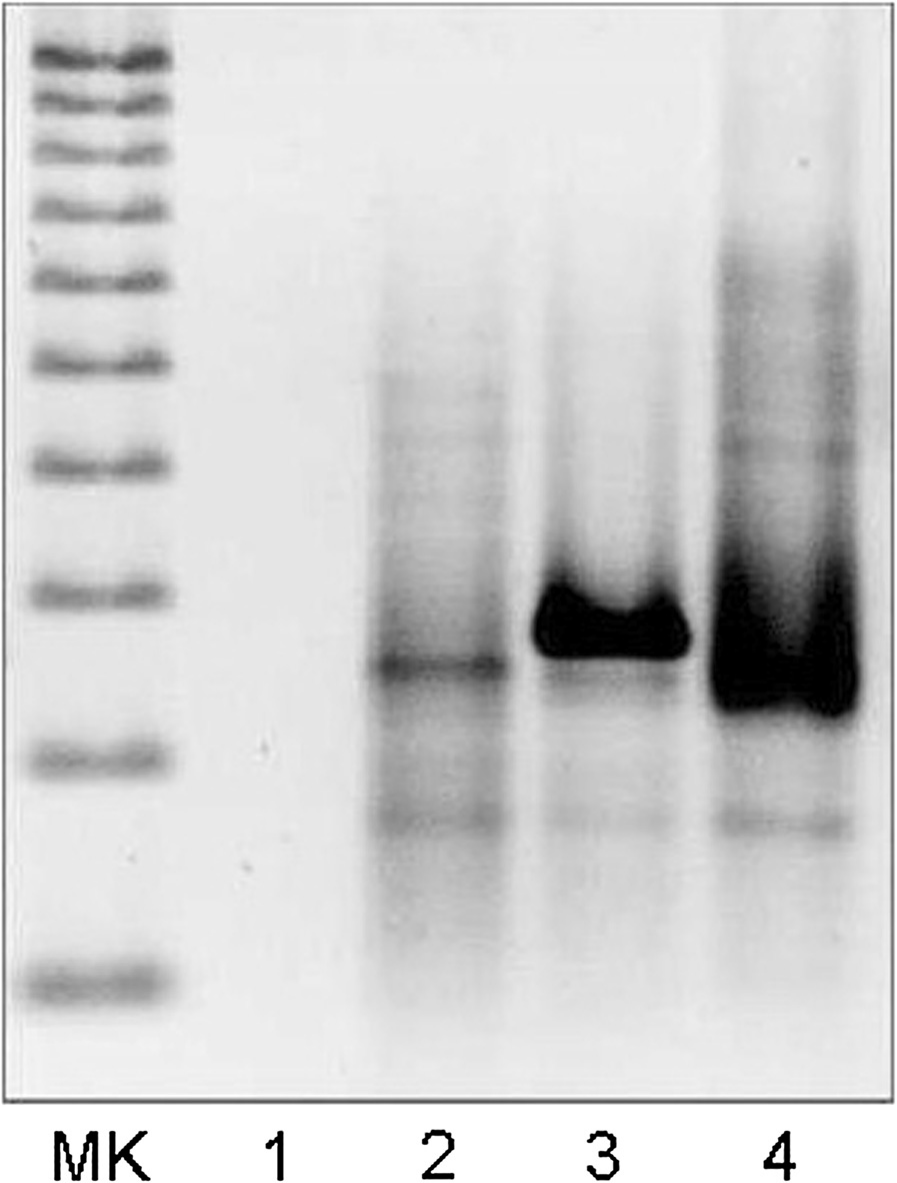
**Agarose gel electrophoresis to detect the rearrangement of the immunoglobulin heavy chain semi-nested polymerase chain reaction products.** Lane MK (marker): Deoxyribonucleic acid (DNA) ladder (100-bp to 1000-bp fragments); lane 1: negative control (no DNA); lane 2: patient sample; lane 3 monoclonal control; lane 4: polyclonal control. A positive β-globin amplification was performed (lane not shown).

Finally, molecular genetic analysis detecting the rearrangement of the FR2/LJH/VLJH region of the immunoglobulin heavy chain was performed and a monoclonal amplicon of approximately 260 base pairs (expected band between 240 and 280 base pairs) was detected, demonstrating malignancy and clonal association of the lymphoma infiltrates in the adenoma (Figure 3). Molecular analyses were carried out as previously described [18,19] and according to the manufacturer’s instructions. Commercial reagents by DAKO Cytomation, Milan, Italy and by Diachem S.r.l., Naples, Italy to perform immunohistochemical and a B clonality assay were used, respectively. All used products are compliant with the requirements of the *in vitro* diagnostic directive 98/79/EC.

The patient underwent full staging for lymphoma. Dawson’s criteria were used in the differential diagnosis between primary colorectal involvement and GI tract involvement secondary to systemic lymphoma [20]. He had no fever, weight loss or night sweats. A physical examination revealed no alteration. There was no lymphadenopathy and hepatosplenomegaly. Blood-cell count, serum biochemistry and immunoglobulins were either within normal limits or negative. A bone marrow biopsy showed no evidence of lymphoma. His chest X-ray was unremarkable. Computed tomography (CT) of his total body revealed no evidence of extraintestinal involvement. A diagnosis of primary DLBCL was made. Ann Arbor stage 1A was established. Subsequently, he was referred to a hematologist for further management. Since the lymphoproliferative lesion was limited and there was no evidence of disseminated disease, and accounting for the advanced age of patient, it was considered inappropriate to perform surgical resection. He did not receive chemotherapy, but he was referred to follow-up with clinical examinations and CT scans at 6-monthly intervals only. He showed no clinical or radiologic recurrence at the time when we wrote this paper (1 year past).

## Discussion

In this article we report a case of primary DLBCL developing within a tubular adenoma with low-grade dysplasia.

Most studies report that lymphomas comprise only 1% to 4% of malignant neoplasms in the GI tract.

Although the GI tract is the most common extranodal location for the development of non-Hodgkin lymphoma (NHL), in adults only 10% to 20% of the primary GI lymphomas occur in the colon. A primary lymphoma of the colon and rectum is an extremely rare entity.

The ileocecal area and ileum are the regions most frequently affected by primary small-intestinal and large-intestinal NHL [16]. Colorectal lymphoma is extremely infrequent, representing less than 0.5% of all primary colorectal neoplasms [16].

Bollen *et al*. report a case of abdominal non-Hodgkin DLBCL in a child [9]. Sikder *et al*. report a case of a 62-year-old Caucasian man showing the presence of a nodule in his rectum that was found to be follicular B-cell lymphoma [10]. Damaj *et al*. report 25 patients with primary follicular lymphoma of the GI tract [11]. Multiple case series show an association between rectal adenocarcinoma and lymphoma because they occur at the same site simultaneously [12,13]. Some studies show that the synchronous diagnosis of colorectal malignancy and lymphoma is rare. Marín García *et al*. report a primary DLBCL of the rectum simulating a rectal adenocarcinoma [15].

Many factors and mechanisms may play a role in the occurrence of synchronous colonic carcinoma and lymphoma [21]. Among these are included environmental agents, immune abnormalities and genetic constitution of the patients [22]. Some authors suggested that the lymphomatous process may be the initial event that compromises the patient’s immune defenses against the development of colon cancer [23].

Little is known about the association of adenoma and lymphomas in the colorectal tract and, to the best of our knowledge, few cases have been reported previously in the literature. Roeb *et al*. present a rare case of synchronous occurrence of a DLBCL initially presenting in a tubular adenoma of the large bowel [16]. Sato *et al*. document a case of T-cell lymphoma associated with a rectal adenoma [17].

In our case, we did not find a specific familial, medical and social history. We have no attractive hypothesis to explain in this patient the synchronous occurrence of dysplasia and lymphoma. It is probable that the factors implicated in the onset dysplasia are the same triggering the carcinogenic process in lymphoproliferative disease. In addition, we consider that the correlation between decreasing immunity and increasing age may be the risk factor for this case.

In general, comprehensive history taking and physical examination may reveal the possible etiologies of some specific lymphoma types and provide information for their further assessment and management.

In this clinical case, a crucial role is represented by stadiation pathology. When a patient is suspected to have a GI lymphoma, staging evaluation for lymphoma is performed, including physical examination; documentation of B symptoms; laboratory evaluation; chest radiograph; CT scan of abdomen, pelvis, and usually chest; bone marrow biopsy; lumbar puncture in lymphoblastic, Burkitt’s lymphoma and DLBCL with positive marrow biopsy; and gallium scan (single photon emission computed tomography) or positron emission tomography scan in large B-cell lymphoma [24].

Although Ann Arbor staging with Musshoff modification is commonly considered the best classification to stage GI lymphomas, the question about the staging strategy remains open due to various available staging systems [2]. GI lymphomas remain the subject of much debate with regard to therapeutic approaches. The role of surgery in primary lymphoma of the rectum is controversial. In most cases of GI primary B lymphomas, surgery has been suggested as the most suitable treatment. The choice of treatment is dependent on the age of patients, clinical scenario, histological subtype, extent and burden of the disease, and comorbidity, besides other factors [2]. This case showed that a primary rectal lymphoma could be treated without surgical resection or chemotherapy. There is strong evidence that the treatment should be defined on the basis of the clinical condition of the patient, evaluating on a case-by-case basis [24]. Lymphoma of the rectum should be considered a different clinicopathological entity with different behaviors, histology and clinical presentation.

The optimal management of primary lymphoma of the colon and rectum has never been determined by randomized trials. In fact, the small number of patients with various histological subtypes and different stage at presentation results in an unclear protocol for the treatment of primary colorectal lymphoma [25].

## Conclusions

Little evidence is available regarding the prognosis of primary colorectal lymphomas. We believe that the case reported here will provide additional information on the history and macroscopic appearance of lymphomas presenting in an unusual location. The epidemiology, clinical presentation, histopathologic subtypes, as well as radiological presentation of GI lymphomas are very important to formulate accurate diagnosis, staging and treatment of the disease with the promising novel techniques. To report additional cases in the future would be helpful in redefining the diagnostic, prognostic and therapeutic approach.

## Consent

Written informed consent was obtained from the patient for publication of this case report and any accompanying images. A copy of the written consent is available for review by the Editor-in-Chief of this journal.

## Competing interests

The authors declare that they have no competing interests.

## Authors’ contributions

TFG performed immunohistochemical and molecular assays, analyzed data, molecular studies and wrote the manuscript; CN, GB and GO contributed to the histological examination; BG contributed to perform histological techniques; GS contributed to perform immunohistochemical techniques; EV analyzed and interpreted the clinical data of the patient; VT analyzed and interpreted the patient data regarding histological and immunohistochemical examination and wrote the manuscript. All authors read and approved the final manuscript. VT and FG contributed equally to this work.

## References

[B1] Wong MTC Eu KW Primary colorectal lymphomas Colorectal Dis 2006 8 586 591 10.1111/j.1463-1318.2006.01021.x 16919111

[B2] Ghimire P Guang-Yao W Zhu L Primary gastrointestinal lymphoma World J Gastroenterol 2011 17 6 697 707 10.3748/wjg.v17.i6.697 21390139 PMC3042647

[B3] Dionigi G Andoni M Rovera F Boni L Villa F Castano P Bianchi V Dionigi R Primary colorectal lymphomas: review of the literature Surg Oncol 2007 16 S169 S171 18024019 10.1016/j.suronc.2007.10.021

[B4] Perry PM Cross RM Morson BC Primary malignant lymphoma of the rectum Dis Colon Rectum 1986 29 821 824 10.1007/BF02555355 2431842

[B5] Freeman C Berg JW Cutler SJ Occurrence and prognosis of extranodal lymphomas Cancer 1972 29 252 260 10.1002/1097-0142(197201)29:1<252::AID-CNCR2820290138>3.0.CO;2-# 5007387

[B6] Henry CA Berry RE Primary lymphoma of the large intestine Am Surg 1988 54 262 266 3364860

[B7] Bairey O Ruchlemer R Shpilberg O Non-Hodgkin’s lymphomas of the colon Isr Med Assoc J 2006 8 832 835 17214096

[B8] Quayle FJ Lowney JK Colorectal lymphoma Clin Colon Rectal Surg 2006 19 49 53 10.1055/s-2006-942344 20011310 PMC2780105

[B9] Bollen P Bourgain C Van Berlaer G Duville L Vandenplas Y Non-Hodgkin lymphoma presenting as a solitary rectal polyp J Pediatr Gasteroenterol & Nutr 2000 31 193 194 10.1097/00005176-200008000-00021 10941976

[B10] Sikder MA Srinivas S Vossough S Primary low-grade lymphoma of the rectum in an asymptomatic patient Pract Gastroenterol 2006 Vol XXX Issue 9

[B11] Damaj G Verkarre V Delmer A Solal-Celigny P Yakoub-Agha I Cellier C Maurschhauser F Bouabdallah R Leblond V Lefrère F Bouscary D Audouin J Coiffier B Varet B Molina T Brousse N Hermine O Primary follicular lymphoma of the gastrointestinal tract: a study of 25 cases and a literature review Ann Oncol 2003 14 623 629 10.1093/annonc/mdg168 12649111

[B12] Song W He YL Han FH Cai SR Peng JJ Rectal non-Hodgkin lymphoma with concomitant rectal adenocarcinoma: a case report and literature review Zhonghua Wei Chang Wai Ke Za Zhi 2011 14 8 617 619 21866456

[B13] Sasaki S Hatanaka K Saha+ra N Uekusa T Hirayama K Shirahata A Ishimaru M Collision tumor of primary malignant lymphoma and adenocarcinoma in the colon: report of a case Surg Today 2010 40 975 981 10.1007/s00595-009-4166-7 20872204

[B14] Deuk YL Seong WH Yeo GC Woo YL Byungmo L Yun KK Synchronous T-cell lymphoma in patient with colon cancer: a case report J Korean Surg Soc 2012 83 60 64 10.4174/jkss.2012.83.1.60 22792536 PMC3392318

[B15] Marín García D Cárdenas Lafuente F Utrilla Ayala Mdel C Galán Jurado MV Jiménez Martín JJ García Ordóñez MA Primary diffuse large B-cell lymphoma of the rectum simulating a rectal adenocarcinoma Gastroenterol Hepatol 2010 33 2 92 98 10.1016/j.gastrohep.2009.08.002 19875198

[B16] Roeb E Rummel M Blau W Etschmann B Gattenlöhner S B-cell lymphoma in a tubular adenoma with high-grade dysplasia: a rare extramedullary manifestation of high-grade diffuse large B-cell lymphoma Endoscopy 2011 43 E344 E345 10.1055/s-0030-1256841 22020719

[B17] Sato H Yasumi K Mizuno Y Ichikawa T Honda K Kuroda M Primary T-cell lymphoma associated with tubulovillous adenoma of the rectum: report of a case Surg Today 2013 43 3 317 320 10.1007/s00595-012-0307-5 22926552

[B18] Nikiforova M His ED Braziel RM Gulley ML Leonard DGB Nowak JA Tubbs RR Vance GH van Deerlin VM Detection of clonal IGH gene rearrangements Arch Pathol Lab Med 2007 131 185 189 17284101 10.5858/2007-131-185-DOCIGR

[B19] van Dongen JJ Langerak AW Brüggemann M Evans PA Hummel M Lavender FL Delabesse E Davi F Schuuring E García-Sanz R van Krieken JH Droese J González D Bastard C White HE Spaargaren M González M Parreira A Smith JL Morgan GJ Kneba M Macintyre EA Design and standardization of PCR primers and protocols for detection of clonal immunoglobulin and T-cell receptor gene recombinations in suspect lymphoproliferations: report of the BIOMED-2 Concerted Action BMH4-CT98-3936 Leukemia 2003 17 2257 2317 10.1038/sj.leu.2403202 14671650

[B20] Dawson IM Cornes JS Morson BC Primary malignant lymphoid tumours of the intestinal tract. Report of 37 cases with a study of factors influencing prognosis Br J Surg 1961 49 80 89 10.1002/bjs.18004921319 13884035

[B21] Nishimura Y Takenaka H Yoshidome K Iwase K Oshima S Tanaka T Primary mesenteric tumor of adult T-cell leukemia/lymphoma: report of a case Surg Today 1994 24 263 267 10.1007/BF02032899 8003870

[B22] Quilon JM Day S Lasker JC Synchronous tumors: Hodgkin disease presenting in mesenteric lymph nodes from a right hemicolectomy for colon carcinoma South Med J 2004 97 1133 1135 10.1097/01.SMJ.0000140827.40139.82 15586613

[B23] Barron BA Localio SA A statistical note on the association of colorectal cancer and lymphoma Am J Epidemiol 1976 104 517 522 984025 10.1093/oxfordjournals.aje.a112324

[B24] Aviles A Neri N Huerta-Guzman J Large bowel lymphoma: an analysis of prognostic factors and therapy in 53 patients J Surg Oncol 2002 80 111 115 10.1002/jso.10103 12173380

[B25] Stanojevic GZ Nestorovic MD Brankovic BR Stojanovic MP Jovanovic MM Radojkovic MD Primary colorectal lymphoma: an overview World J Gastrointest Oncol 2011 3 1 14 18 10.4251/wjgo.v3.i1.14 21267399 PMC3026053

